# Insights Into the Host Contribution of Endocrine Associated Immune-Related Adverse Events to Immune Checkpoint Inhibition Therapy

**DOI:** 10.3389/fonc.2022.894015

**Published:** 2022-07-14

**Authors:** Adrian Chye, India Allen, Megan Barnet, Deborah L. Burnett

**Affiliations:** ^1^ Immunology Division, Garvan Institute of Medical Research, Darlinghurst, NSW, Australia; ^2^ St Vincent’s Clinical School, Faculty of Medicine, University of New South Wales, Darlinghurst, NSW, Australia; ^3^ Department of Medical Oncology, The Kinghorn Cancer Centre, Darlinghurst, NSW, Australia

**Keywords:** cancer, immune checkpoint inhibitor (ICI), immune related adverse events (irAE), immunotherapy, autoimmunity, genetic biomarkers

## Abstract

Blockade of immune checkpoints transformed the paradigm of systemic cancer therapy, enabling substitution of a cytotoxic chemotherapy backbone to one of immunostimulation in many settings. Invigorating host immune cells against tumor neo-antigens, however, can induce severe autoimmune toxicity which in many cases requires ongoing management. Many immune-related adverse events (irAEs) are clinically and pathologically indistinguishable from inborn errors of immunity arising from genetic polymorphisms of immune checkpoint genes, suggesting a possible shared driver for both conditions. Many endocrine irAEs, for example, have analogous primary genetic conditions with varied penetrance and severity despite consistent genetic change. This is akin to onset of irAEs in response to immune checkpoint inhibitors (ICIs), which vary in timing, severity and nature despite a consistent drug target. Host contribution to ICI response and irAEs, particularly those of endocrine origin, such as thyroiditis, hypophysitis, adrenalitis and diabetes mellitus, remains poorly defined. Improved understanding of host factors contributing to ICI outcomes is essential for tailoring care to an individual’s unique genetic predisposition to response and toxicity, and are discussed in detail in this review.

## Introduction

Monoclonal antibodies that block specific immune checkpoints enable durable anti-cancer response in a subset of patients with previously futile prognoses. Drugs targeting Programmed Cell Death Protein-1 (PD-1), its ligand (PD-L1) and Cytotoxic T-lymphocyte Antigen-4 (CTLA-4) are now considered part of standard of care for treatment of multiple cancers. Both targets represent immune checkpoints that contribute to physiological immune tolerance (as summarized here (1)). Despite remarkable response to PD-1/PD-L1 and/or CTLA-4 blockade in some patients, however, a substantial proportion do not gain benefit and mechanisms underlying individual variation in response are incompletely understood.

Immune-related adverse events (irAEs) are autoimmune sequelae of ICI therapy that can affect any organ in the body. Multiple endocrine irAEs including thyroiditis, hypophysitis, diabetes mellitus and adrenalitis, have been recognized with the use of ICI. Endocrinopathies are amongst the most common irAEs. Partly due to the large reserve capacity of many endocrine organs, they are distinct in their tendency to be diagnosed at the point of organ failure. In contrast, most non-endocrine irAEs are detected based on early clinical indications of inflammation, such as dyspnea or diarrhea. As such, the management of endocrine irAEs typically includes life-long hormone replacement (2). While non-endocrine irAEs are often treated with glucocorticoids, there is no clear evidence that immunosuppression is beneficial in preventing progression of endocrinopathy (2).

Successful checkpoint inhibition in cancer treatment requires a fundamental shift in immune homeostasis to a state of reduced immune tolerance. ICI therapy is thus inherently linked to irAEs, because lowering immune tolerance to cancer inextricably lowers tolerance to self. Through this lens, manifestation of irAEs may be a marker of successful immune activation. Accordingly, the occurrence and severity of irAEs, particularly those of endocrine origin, have been linked to improved cancer outcomes in studies that have corrected for immortal time bias in several tumors (3–6).

Significantly, many irAEs are clinically and pathologically indistinguishable from autoimmune disease associated with inherited variation in relevant checkpoint genes. Penetrance of irAEs following pharmacologic blockade of a checkpoint, however, is not equivalent to that of primary genetic blockade of the same gene (7, 8). Intriguingly, pharmacologic blockade in some cases causes more frequent endocrinopathy than genetic blockade, and in some cases less (**Table 1**). Clinical autoimmunity may manifest when acquired mutations layer on inherited mutations, stochastically bypassing tolerance to precipitate disease (19–22). Pharmacologic blockade of a checkpoint (such as PD-1) may be equivalent to an acquired functional genetic mutation, serving as an additional ‘switch’ weighing in favor of autoimmunity. Improved understanding of the mechanism for drug-induced irAEs and their similarities and differences to genetic autoimmune disease, therefore, has potential to improve patient selection and cancer outcomes.

**Table 1:** Comparison of autoimmune manifestations in genetic deficiency versus pharmacological inhibition of CTLA-4 and PD-1/PD-L1.

Autoimmune manifestation	Genetic *CTLA4* deficiency (9, 10)	CTLA-4 inhibitor (11–14)	Genetic *PDCD1* deficiency (15)	PD-1 or PD-L1 inhibitor (11, 16–18)
Endocrine involvement	33%	7-37%	present	9.4-23%
Thyroiditis	5-15%	1.5-9%	present	10.8-20.4%
Hypophysitis	<1%	2.3-18%	not detected	1.8-2.2%
Diabetes mellitus	0-5%	not detected	present	0.4-2%
Adrenalitis	not detected	1.3-1.4%	not detected	1-2%
Skin involvement	21-56%	43-63%	present	5.3-44.5%
Gastrointestinal involvement	59-78%	29-45%	not detected	3.9-25.2%
Liver involvement	12%	3.8-25%	present	1.8-9.1%
Respiratory involvement	57-68%	1.1-14%	present	1.3-4.7%
Neurological involvement	29%	3-5%	not detected	not detected
Autoimmune cytopenia	62-63%	not detected	present	not detected
Hypogammaglobulinemia	76-84%	not detected	not detected	not detected
Lymphoproliferation	73%	not detected	present	not detected

In this review, we explore physiological mechanisms of immune tolerance that are leveraged in cancer immunotherapy and examine ICI-induced toxicity and analogous genetic disease, with a focus on endocrinopathies and host contribution to irAE response.

## Physiological Immune Tolerance: Essential to Immune Homeostasis

Understanding tolerance in different cell types and physiological settings is an important foundation to understanding ICI response and toxicity. The immune system balances a challenging equilibrium of mounting appropriate response to pathogens whilst tolerating self and non-self commensals. Differing mechanisms of tolerance induction and maintenance for T and B cells, in central (bone marrow, thymus) and peripheral (spleen, lymph nodes) lymphoid organs, are summarized below. The majority of cancer immunotherapy research to date has focused on the role of T-cells, however B-cells play an important role in analogous primary autoimmune disease. Furthermore, auto-antibodies are a feature of many endocrine-related irAEs and may be key to understanding pathogenesis.

### T-Cell Tolerance

Central T cell tolerance is established during T cell development in the thymus. Thymocytes express a repertoire of T cell receptors (TCRs), and undergo a process of positive and negative selection (23). During positive selection, thymocytes with TCRs that have sufficient affinity for peptide-Major Histocompatibility Complex (MHC) are selected for survival, while during negative selection thymocytes that bind to self-peptide are signaled to apoptose (24). T cells with a TCR that has sufficient affinity to self-peptide MHC molecules receive a survival signal and are released as naïve cells into the periphery, while T cells with insufficient MHC molecules undergo apoptosis (24). T cells with potentially hazardous self-reactive TCRs that have strong reactivity to self-peptides undergo clonal deletion or receptor editing in the thymus to reduce their reactivity (25). However, central tolerance is only partially effective in eliminating self-reactive T cell clones, allowing release of self-reactive T cell clones into the periphery (26).

Self-reactive naïve T cells that escape central tolerance are maintained by a number of peripheral T cell tolerance mechanisms including quiescence, ignorance, anergy, exhaustion, senescence and death (27). Quiescence is an active process that maintains naïve T cells at a lower metabolic state, in the G0 stage of the cell cycle, agnostic to TCR stimulation (28). Clonal ignorance is a mechanism maintaining self-reactive T cells in an inactive state despite the presence of self-antigen (29–31). T cell ‘anergy’ can result from unbalanced or deficient TCR stimulation without engagement of costimulatory receptors (32–34). When T cells are successfully stimulated and reach the effector stage, T cell exhaustion may arise, characterized by reduced effector function and sustained inhibitory receptor expression. In the context of chronic infections, T cell exhaustion avoids pathogenic inflammation and autoreactivity (35). T cells may eventually senesce and die, arresting growth when they reach their replicative potential or are exposed to various stressors (36). Additionally, peripheral deletional tolerance checkpoints serve a critical role in pruning the repertoire of peripheral T cells and terminating deleterious immune responses, preventing T cell mediated immunopathology (37).

### B-Cell Tolerance

Highly reactive B Cell Receptors (BCRs) also experience negative selection and are actively eliminated in the bone marrow by central tolerance mechanisms including clonal deletion (38) and receptor editing (39, 40). Unlike T cells however, the extent to which B cells undergo positive selection to self-antigen in the bone marrow is still not well defined. Some mildly and moderately self-reactive B cells have been shown to undergo positive selection into the mature repertoire (41). Regardless of the mechanism, central B cell tolerance results in a significant proportion of auto reactive BCRs surviving and exiting the bone marrow into the periphery (42, 43). Assessment of mature peripheral B cells in healthy human controls show that 5-20% of cells encode for antibodies with poly- or self-reactivity (44). Self-reactive B cells tend to exist in a functionally silenced state of anergy (45). The benefit of preserving these potentially harmful auto-reactive cells was only recently shown to be the ability for “auto-antibody redemption”, wherein BCRs mutate away from self-reactivity to enable production of antibodies with high foreign antigen specificity (46–50).

## Pathological Immune Tolerance: Immune Evasion by Cancer Cells

“Successful” cancer cells achieve unchecked proliferation by exploiting physiological mechanisms of immune tolerance to evade detection and/or destruction by the host immune system. These include downregulation of antigen-presenting machinery including MHC-1 (51), overexpression of immune-inhibitory molecules such as PD-L1 (52), induction of an immunosuppressive tumor microenvironment enriched for regulatory T cells (Tregs) and myeloid-derived suppressor cells (53–55) and secretion of directly immunosuppressive factors such as IL-10, Transforming Growth Factor-β (TGF-β), gangliosides, prostaglandin E2, and vascular endothelial growth factor (VEGF) (56–60).

Tregs play a critical role in tolerance to cancer cells through suppression of cytotoxic CD8+ T-cell proliferation, contributing to immune escape. Tregs express high levels of multiple checkpoint receptor molecules including CTLA-4, PD-1, T cell immunoreceptor with Ig and ITIM domains (TIGIT), Lymphocyte-Activation Gene 3 (LAG-3) and T-cell immunoglobulin domain and mucin domain 3 (TIM3) (61, 62).

## Immune Checkpoint Inhibitors

Monoclonal antibodies targeting CTLA-4 and PD-1/PD-L1 are now considered part of standard of care in multiple cancer types, including melanoma, Non-Small Cell lung cancer (NSCLC), renal cell carcinoma, head and neck cancer, urothelial cancer, Merkel cell carcinoma, mesothelioma, and Hodgkin lymphoma.

CTLA-4 is a negative co-inhibitory receptor of T-cell activation and proliferation, playing a critical role in the maintenance of self-tolerance (63). It is constitutively upregulated in Tregs and transiently upregulated on the cell surface of conventional T cells following the engagement of co-stimulatory molecule CD28 with its ligands, CD80 and CD86, in order to curtail T cell responses (64). CTLA-4 has several mechanisms of action including competitively binding CD80 and CD86 and reducing CD28-dependent co-stimulation (65–67). Additionally, CTLA-4 is thought to strip the CD80 and CD86 ligands from APCs through a process of trans-endocytosis, further reducing potential for CD28 co-stimulation (65). It has been postulated that CTLA-4 may also directly restrain T-lymphocytes through down-regulating IL2 expression, as well as repression of T-cell proliferation (68).

PD-1 is a co-inhibitory receptor expressed on the surface of activated T-cells (including tumor infiltrating lymphocytes), B cells, NK cells, monocytes and dendritic cells (64, 69, 70). Ligation of PD-1 with its ligands, PD-L1 and PD-L2, activates inhibitory signaling cascades associated with exhaustion, resulting in the downregulation of T-cell response (69). PD-1 suppression of T cell activity occurs primarily within the peripheral tissues and tumor microenvironment (71).

The incidence and pattern of endocrine irAEs differs significantly between the major classes of checkpoint inhibitors, reflective of differing mechanisms of action (**Table 1**). ICI-induced thyroiditis is more frequent after PD-1 compared with CTLA-4 blockade (10-20.4% versus 1.5-9%). Whereas ICI-induced hypophysitis and colitis are more common following CTLA-4 compared with PD-1 inhibition (2.3-18% versus 1.2-2.2% and 29-45% versus 3.9-25% respectively). Combination checkpoint blockade, targeting both CTLA-4 and PD-1, increases rate and severity of irAEs, with tendency to earlier events and multi-organ autoimmunity (72, 73).

## Endocrine irAEs: Prevalence and Significance

Accurate predictive models for type and severity of irAEs are lacking, although several potential biomarkers are being studied involving immune cells (74, 75), cytokines (76), autoantibodies (77), immunogenetics (78, 79) and microbiome (80, 81). Time of onset is also unpredictable; most irAEs develop within the first few weeks following commencement of an ICI, however they can manifest years after treatment initiation (82). IrAEs occur in up to 80% of patients receiving combination treatment (83), and are predominantly mild (CTCAE grade 1-2) (84), with less than 1% resulting in death (85).

Endocrinopathies are a subset of irAEs that affect endocrine organs, including thyroiditis, hypophysitis, diabetes mellitus and adrenalitis (86). Distinct to non-endocrine irAEs, endocrine-related irAEs are frequently irreversible, do not usually prompt treatment discontinuation and often require lifelong hormone replacement therapy (87). While numerous studies have examined the relationship between irAEs and treatment outcomes, most do not account for immortal time bias; an adjustment for shorter time on drug and less time available to develop irAEs for non-responders. Landmark analyses avert this bias and also support a correlation of irAEs with outcomes, particularly those involving endocrine organs and skin for patients with NSCLC and melanoma (3–6). The correlation of irAEs with survival in many settings suggests a possible shared driver of systemic and anti-cancer immune activation.

### ICI-Induced Thyroiditis

ICI-induced thyroiditis is common in patients treated with ICI, with an incidence of up to 20% for single agent anti-PD-1 therapy (16). The clinical phenotype is painless thyroiditis, most often characterized by transient thyrotoxicosis followed by euthyroidism or progression to hypothyroidism without preceding thyrotoxicosis (88, 89). The true incidence of thyroiditis is likely underreported, in part because clinical trials define ICI-mediated hypothyroidism and hyperthyroidism as separate entities rather than a single pathology.

ICI-induced thyroiditis has much clinical and biochemical overlap with spontaneous autoimmune thyroid diseases (AITD), including Hashimoto’s thyroiditis and its variant form, subacute painless thyroiditis (also known as silent thyroiditis or subacute lymphocytic thyroiditis) (90). In AITD, antigen presenting cells and helper CD4+ T cells activate CD8+ T cells specific for thyroid parenchyma, causing direct tissue injury and thyroid antigen exposure. B cell activation from thyroid tissue leads to expression of anti-thyroid autoantibodies to thyroglobulin (Tg) and thyroid peroxidase (TPO) (91). It is unclear whether ICI-induced thyroiditis reflects exacerbation of an evolving AITD, or has a distinct mechanism with the shared endpoint of auto-reactive B cells. Anti-PD-1-induced destructive thyroiditis in preclinical modelling, however, was completely prevented by depletion of CD4+ T cells, partially prevented by depletion of CD8+ T cells but not affected by depletion of CD20+ B cells (92). This supports a more T-cell dominant process at play.

In patients with AITD, PD-1 positive T cells are significantly expanded in both the periphery and intrathyroidal lymphocytes compared to patients with (non-autoimmune) multi-nodular goiter (MNG) (93). PD-L1 positive parenchymal thyroid follicular cells are present in most AITD glands, but rarely in MNG glands, and have been hypothesized to serve as a peripheral tolerance mechanism induced to avoid recognition by self-reactive T lymphocytes (93).

Thyroid auto-antibodies develop in up to 70% of patients with ICI-induced thyroiditis (77). It is unclear, however, if these are generated in response to thyroid antigen released during destructive thyroiditis, or if ICI reactivates dormant thyroid auto-antibodies, therein initiating thyroiditis. Patients with pre-existing anti-thyroglobulin and anti-thyroid peroxidase antibodies have an increased propensity to ICI-induced thyroiditis compared with patients who do not harbor these autoantibodies (90, 94, 95). Moreover, there is shared genetic predisposition to AITD and ICI-induced thyroiditis, highlighting the direct contribution of host predisposition (96). Many patients who develop thyroid irAEs, however, do not have detectable thyroid auto-antibodies at baseline or following diagnosis of ICI-induced thyroiditis, suggesting an alternative pathogenesis distinct from AITD (97).

### ICI-Induced Hypophysitis

ICI-induced hypophysitis is inflammation of the pituitary gland resulting in the decreased production of one or more pituitary hormones. It is more common following CTLA-4 blockade compared with PD-1 blockade (3.2% and 0.4% respectively), with incidence up to 6.4% in combination CTLA-4/PD-1 treatment (86). Symptoms may include headache, fatigue, anorexia or myalgias, but it is often diagnosed during investigation of low cortisol on routine bloods or on presentation in adrenal crisis. The diagnosis may be supported by radiographic pituitary enlargement. A uniform diagnostic criterion does not exist, therefore existing literature may inaccurately report case frequency (98).

There are key distinctions in the clinical phenotype between hypophysitis caused by CTLA-4 compared with PD-1 inhibitors. Firstly, in time of onset. CTLA-4 induced hypophysitis usually presents within weeks of ICI commencement (mean 9 weeks), whereas anti-PD-1-induced disease is more variable, but tends to occur later (mean 26 weeks) (99). Time to onset of disease in combination therapy falls between these two timeframes. Secondly, in symptomatology. Headache is more frequent in patients treated with CTLA-4-inhibitor or combination therapy, while the predominant symptoms of PD-1/PD-L1-inhibitor induced hypophysitis are fatigue, anorexia and myalgias (99, 100). Thirdly, axes involvement. Patients with anti-PD-1 related hypophysitis often have isolated ACTH-adrenal axis insufficiency, while CTLA-4-inhibitor related hypophysitis tends to involve multiple pituitary axes. Finally, in radiographic changes. Pituitary gland enlargement on MRI was almost universal in one multi-center review for patients with hypophysitis following CTLA-4 therapy, while this was only seen in a minority of cases (28%) resulting from anti-PD-1 therapy (99).

The underlying mechanism of ICI-mediated hypophysitis is largely unknown. One theory is that immune-related hypophysitis occurs more frequently in patients treated with CTLA-4 inhibitors because it directly targets pituitary gland cells, which express CTLA-4 and may activate antibody-dependent cell-mediated cytotoxicity and complement-mediated direct injury (101–103). In a murine model, CTLA-4 blockade resulted in complement deposition on endocrine cells including the pituitary (101). A human autopsy series showed disproportionately high CTLA-4 antigen expression within the pituitary of patients who had developed ICI-induced hypophysitis (104). The precise molecular basis for anti-PD-1 hypophysitis is unknown.

### ICI-Induced Diabetes Mellitus

ICI-induced diabetes mellitus (ICI-DM) is defined by severe and persistent insulin deficiency caused by pancreatic β-islet cell failure following treatment with ICI (105). ICI-DM is rare, and occurs almost exclusively with anti-PD-1/PD-L1 therapy. A meta-analysis of 101 studies demonstrated the incidence of ICI-DM to be 0.4% for pembrolizumab, 2.0% for nivolumab, with no cases for patients treated with ipilimumab (11). A systematic review of the existing literature identified only 90 patients with ICI-induced DM, and observed that most patients were treated with anti-PD-1/PD-L1 monotherapy (71%) or anti-PD1/PD-L1 in combination with CTLA-4 blockade (15%), while anti-CTLA-4 alone accounted for only 3% of the patients (106).

The clinical presentation of ICI-DM is similar to that of classic type 1 diabetes mellitus (T1DM), ranging from asymptomatic hyperglycemia, polyuria, polydipsia, and fatigue, to life-threatening diabetic ketoacidosis. ICI-DM onset is also highly variable, with reported median time to diagnosis of between 7 to 17 weeks post treatment commencement, with potential to present years later (107, 108). As with other endocrine irAEs, ICI-DM is generally diagnosed at point of organ failure, and almost always requires life-long insulin replacement therapy (87).

ICI-DM is presumed to be caused by immune-mediated destruction of pancreatic β-cells, however may be pathologically distinct from T1DM (109). Analysis of pancreas tissue from a patient who developed ICI-DM following combination therapy showed marked peri-islet infiltration of CD8+ T cells, with very few residual pancreatic β cells. Destruction of β-cells was noted to be more severe than most cases of classic T1DM, and may reflect mechanistic differences (109). ICI-DM and classic T1DM also differ in prevalence of auto-antibodies. More than 90% of patients with T1DM have at least one of four common β-cells autoantibodies; to insulin, GAD (glutamic acid decarboxylase), ZnT8 (zinc transporter 8) or IA-2 (insulinoma-associated-2) (110). In contrast, only 50% of patients with ICI-DM are found to have a relevant autoantibody, with anti-GAD65 comprising the majority of these (105, 107). This may suggest that either autoantibodies play less of a role in the pathogenesis of ICI-DM compared to primary T1DM, or potentially that the autoantibodies still exist, but are sequestered in tissue.

### ICI-Induced Adrenalitis

ICI-induced adrenalitis is a rare endocrine irAE and is defined by adrenal gland inflammation leading to adrenal cortex failure to generate hormones, including glucocorticoids, mineralocorticoids and sex steroids. A meta-analysis revealed an incidence of 0.7% across studies of monotherapy with anti-PD-1/PD-L1 or anti-CTLA-4, but up to 4.2% in studies of combination therapy (86). This shows some discordance with a WHO pharmacovigilance database review, which demonstrated that the majority of patients diagnosed with adrenalitis had been treated with PD-1 monotherapy (56%), and less commonly with CTLA-4 monotherapy (23.6%) or combination therapy (17.9%) (111). Primary adrenal insufficiency may be diagnosed on routine bloods showing reduced cortisol with appropriate elevation of pituitary-derived ACTH, but may present clinically with fatigue, orthostatic hypotension, or adrenal crisis (112). After acute management, long-term corticosteroid (with or without mineralocorticoid) replacement is usually required (113).

ICI-adrenalitis is presumed to be caused by immune-mediated destruction of the adrenal cortex (111). In published case reports, antibodies to 21-hydroxylase were detected in a handful of cases (114, 115), which may suggest a B-cell mediated process. The analogous autoimmune condition of primary adrenal insufficiency (Addison’s disease), is also characterized by antibodies against the steroidogenic enzymes; most commonly 21-hydroxylase, and less frequently a side-chain cleavage enzyme or 17-alpha-hydroxylase (116).

## Pharmacological Inhibition Compared With Genetic Inhibition of Immune Checkpoints

Pharmacological inhibition of CTLA-4 and/or PD-1/PD-L1 has significant overlap, but also important differences, to autoimmune manifestations seen in humans with inborn errors of the corresponding genes, *CTLA4* and *PDCD1* (**Table 1**).

Mice homozygous for *ctla-4* loss of function develop lymphoproliferative symptoms in multiple organs and die within weeks, but mice with *ctla4* haploinsufficiency (one functional allele retained) remain healthy (117–119). In humans, inherited genetic heterozygosity for *CTLA4* inactivating mutations results in immunodeficiency and autoimmunity (9, 10, 117, 120). In the largest study of *CTLA4* haploinsufficiency in humans, 133 patients were characterized (10). The median age of onset of disease was 11 years and there was clinical penetrance (manifestation of disease) in 67% (10). The predominant clinical phenotypes included hypogammaglobulinemia (84%), lymphoproliferation (73%) and organ-specific autoimmunity such as hematological autoimmune cytopenia (62%), respiratory (68%), and gastrointestinal (59%) or neurological manifestations (29%) (10) (**Table 1**). Some auto-immune sequelae of germline *CTLA-4* deficiency are almost indistinguishable from toxicity following its pharmacologic blockade, such as gastrointestinal inflammation (10, 12, 13). One factor contributing to observed differences may be distinct temporal dynamics between in-born genetic loss of *CTLA-4* compared with acquired pharmacologic blockade, usually later in life. Mouse models of conditional CTLA-4 inhibition later in life, however, do not more closely resemble anti-*CLTA-4* induced irAEs, suggesting other differences at play (121).

Key discrepancies between genetically and pharmacologically driven disease highlight the need to further understand both pathways. Firstly, clinical apparent autoimmunity, in general, appears to be more prevalent in humans with *CTLA-4* haploinsufficiency compared with patients receiving *CTLA-4* pharmacological blockade (10, 12, 13). This supports the notion that irAEs associated with CTLA-4 blockade are dose-dependent, and the monoclonal antibodies may only partially inhibit *CTLA4* function leading to incomplete constellation of symptoms. Indeed, severe, life-threatening autoimmune toxicities are more frequent with higher doses of ipilimumab supporting this dose-dependent effect (122). Secondly, and somewhat counter to this, certain organ toxicities are significantly more common with CTLA-4 drug blockade compared with *CTLA-4* germline haploinsufficiency. For example, severe hypophysitis is reported in up to 5% of patients treated with ipilimumab but is extremely rare (<1%) in genetic *CTLA4* haploinsufficiency (10, 13) (**Table 1**). Finally, certain manifestations of primary *CTLA4* deficiency are not seen in the drug-induced setting, significantly immunodeficiency, which is contributed to by autoantibody mediated cytopenia, lymphadenopathy and splenomegaly (9, 120, 123). The reason for this discord is not well understood.

In contrast to *CTLA4*, germline deficiencies in *PD-1* more frequently lead to single organ effects, most commonly involving endocrine organs. Here, exogenous blockade appears to more closely match germline inactivation (124–126). Mice deficient in PD-1 initially develop normally but go on to manifest strain-dependent autoimmunity and heightened inflammation during infections (127–129). PD-1 deficiency also accelerates the onset and frequency of organ-specific endocrine autoimmunity such as T1DM in predisposed mouse models (130).

In humans, homozygous loss of function mutations in the gene encoding PD-1, *PDCD1*, is exceedingly rare (15). In one case study, homozygous frameshift (loss of function) mutation resulted in lymphoproliferative autoimmunity, autoimmune thyroiditis, T1DM, juvenile idiopathic arthritis, and abdominal tuberculosis. The patient died of pneumonitis at age 10 years (15). Though complete loss of *PDCD1* is rare in humans and difficult to study, partial deficiency shows significant overlap with drug-induced irAEs (**Table 1**).

Further investigation is needed to explore similarities and discrepancies between genetic and drug-induced autoimmunity. As discussed above, timing and duration of exposure to pathway blockade is likely to play a role. Inherited genetic abnormalities may only manifest as autoimmunity when accumulation of acquired mutations leads to a break in tolerance (20). Treatment with anti-CTLA-4 and/or anti-PD-1 can be considered akin to an acquired mutation in terms of onset later in life, however is distinct from an acquired mutation in that blockade is maintained for a fixed period only. Comparison with analogous genetic disease is valuable because it can help elucidate mechanism, however genetic predisposition is likely to be just one of many factors contributing to outcome. Environmental factors including the microbiome are likely to play a major role (80, 81), but are beyond the scope of this review.

## Shared Genetic Predisposition to Autoimmunity, irAE and ICI Response

Recent genetic association studies have revealed that polymorphisms in *CTLA4* may modulate both autoimmunity and response and toxicity to ICIs (131, 132). Specific gene associations with autoimmune disease are outlined in **Table 2**. Many of these Genome-wide association studies (GWAS) single nucleotide polymorphisms (SNPs) highlight the dialectic nature of T-cell immune regulation in cancer and autoimmunity - the allele for cancer risk is often the opposite to that for autoimmune disease (132, 134, 157, 158, 166–170). Systemic testing for common autoimmune risk loci could provide a pathway for personalized biomarkers for ICI response and toxicity (171).

**Table 2:** Germline *CTLA4* and *PDCD1* gene polymorphisms are associated with autoimmune diseases.

Autoimmune disease	Gene polymorphism	Ethnic Group	References
Autoimmune thyroid disease including Grave’s disease and Hashimoto’s thyroiditis	*CTLA4*	United Kingdom Chinese Japanese Brazilian Indian	(117, 133) (134–137) (138) (139) (140)
Type 1 Diabetes Mellitus	*CTLA4* *PDCD1*	Dutch European United Kingdom Chinese Tunisian Italian Danish European Chinese	(141) (117) (142) (143) (144) (124) (145)
Autoimmune adrenal insufficiency	*CTLA4*	European	(146)
Vitiligo, in combination with other autoimmune disease	*CTLA4*	United Kingdom	(147)
Pemphigus vulgaris and foliaceus	*CTLA4*	Spanish European Serbian European Brazilian	(148) (148, 149) (150)
Coeliac disease	*CTLA4*	Dutch European European French Caucasian	(141) (151) (152)
Rheumatoid arthritis	*CTLA4* *PDCD1*	United Kingdom North America European/Mexican Chinese	(133) (153) (154) (155, 156)
Myasthenia Gravis	*CTLA4*	Chinese Swedish European	(157–162) (163) (149)
Systemic lupus erythematosus	*CTLA4* *PDCD1*	Korean Cretan/Greek European	(143) (164)
Sjogren’s syndrome	*CTLA4*	Australian Caucasian	(165)

Germline polymorphisms in *CTLA4* have also been shown to influence susceptibility to irAEs. Refae et al. investigated a cohort of 94 patients with advanced cancer treated with anti-PD-1/PD-L1 and demonstrated that SNPs within a range of genes including *UNG*, *IFNW1*, *CD274* and *IFNL4* were predictive of irAEs corresponding to that gene function (78). Whole genome sequencing of 479 patients with bladder cancer treated with anti-PD-L1 therapy showed that high polygenic risk scores for vitiligo and psoriasis were associated with increased risk of skin-related irAEs (7).

A key question is whether the genetic risk for classical T1DM overlaps with ICI-DM. Polymorphisms of multiple genes are known to influence risk of T1DM, with the HLA-DQ, HLA-DR alleles having the largest effect, but also non-HLA genes such as *PTPN22* and *CTLA-4* (172). HLA polymorphisms linked to classic T1DM class II haplotypes – HLA-DR3-DQ2 and DR4-DQ8 in European populations, and DR4-DQ4 and DR9-DQ9 in Asian populations, have been demonstrated to be overrepresented patients with ICI-DM (106). Only one ICI-DM case has been evaluated for multiple HLA and non-HLA risk genes as part of a T1DM genetic risk score (GRS); this case had a GRS below the 5^th^ percentile for T1DM (173), suggesting that ICI-DM genetic risk factors may be distinct from T1DM. Overall, HLA susceptibility haplotypes for T1DM may predispose to the development ICI-DM, but further studies are needed to understand the genetic contribution of HLA and non-HLA genes.

There is also known genetic susceptibility to spontaneous AITD, within which certain at-risk genes may also be certain at-risk genes may also be shared with ICI-induced thyroiditis. Data suggests there is an association of AITD with certain HLA alleles, particularly HLA-DR3, and with polymorphisms within *CTLA4* and *CD40* (174). GWAS have demonstrated key susceptibility genes implicated in both immune regulation and increased risk of ICI-induced thyroiditis, including *CD69*, *CTLA4*, *PTPN22* and *LPP* (96). These loci and others were combined to calculate a polygenic risk score that could identify a cohort of patients with more than 6-fold increased risk of ICI-induced thyroiditis. Furthermore, germline susceptibility to AITD disease was found to be associated with improved survival, in a cohort of patients with triple-negative breast cancer treated with combination anti-PD-L1 inhibitor and chemotherapy (96).

## Conclusion

Immune checkpoint inhibitors are now established as a pillar of cancer treatment, alongside chemotherapy, radiotherapy and surgery. Understanding mechanism of response and non-response to the drugs is paramount to broadening their potential for clinical benefit. Investigating drivers of ‘off-target’ effects, specifically irAEs, is key to this analysis, because anti-self responses are inextricably linked with anti-cancer responses in many settings due to lowering of immune tolerance thresholds. Furthermore, many irAEs are clinically and biologically analogous to genetic disease, for which we have significant insight into drivers and mitigating factors.

Autoimmunity of endocrine organs are common both as primary disease and as ICI-induced toxicity in patients treated for cancer. Endocrine autoimmunity driven by genetic variation in checkpoint genes can mimic drug-induced irAEs, however key differences exist in the pathophysiology for each. Both primary and drug-induced endocrine disease may involve underlying genetic susceptibility as part of a multifactorial pathogenesis. Further investigation into shared drivers of irAEs with analogous primary disease may lead to predictive models for genetic predisposition to ICI-induced toxicity. Furthermore, the correlation between irAEs and anti-cancer response highlights the potential for these genetic drivers to serve as novel therapeutic targets, with the caveat that ensuing toxicity may need aggressive management.

Current predictive markers (such as tumor and immune cell PD-L1 expression) can enrich for responders to immunotherapy but provide little insight into variation in cancer response or toxicity for the individual patient. There is increasing recognition that host factors, including genetic predisposition to both autoimmunity (for example to thyroid disease) and irAEs (for example to psoriasis and vitiligo), can be predictive of checkpoint inhibitor treatment outcomes. Genetic testing for common autoimmune risk loci could form part of a comprehensive personalized biomarker for ICI response and toxicity, leading to improved treatment selection and toxicity management for individual patients.

There is much to learn from host factors that contribute to maintenance and breakdown of physiological immune tolerance. Shared drivers of clinical autoimmunity and ICI-induced irAEs are pivotal to this analysis and have the potential to progress patient outcomes in cancer treatment significantly.

## Author Contributions

AC, IA, MB and DB jointly wrote the manuscript. All authors contributed to the conception, design, and interpretation of the manuscript, and reviewed and wrote the final paper.

## Funding

This work was supported by National Health and Medical Research Council (NHMRC) (Grants 10375), and by Bioplatforms Australia and The Kinghorn Foundation as part of the Australian Exceptional Responders Program (Grants PR10285).

## Conflict of Interest

The authors declare that the research was conducted in the absence of any commercial or financial relationships that could be construed as a potential conflict of interest.

## Publisher’s Note

All claims expressed in this article are solely those of the authors and do not necessarily represent those of their affiliated organizations, or those of the publisher, the editors and the reviewers. Any product that may be evaluated in this article, or claim that may be made by its manufacturer, is not guaranteed or endorsed by the publisher.
